# Functionality and quality of life of patients with unilateral lymphedema of a lower limb: a cross-sectional study

**DOI:** 10.1590/1677-5449.006618

**Published:** 2019-05-07

**Authors:** Barbara Cristina de Sousa Pedrosa, Juliana Netto Maia, Ana Paula de Lima Ferreira, Maria das Graças Rodrigues de Araújo, Eduardo José Nepomuceno Montenegro, Fernando Leonel da Silva, Célia Maria Machado Barbosa de Castro, Maria do Amparo Andrade

**Affiliations:** 1 Universidade Federal de Pernambuco – UFPE, Laboratório de Imunopatologia Keizo Asami – LIKA, Recife, PE, Brasil.; 2 Universidade Federal de Pernambuco – UFPE, Departamento de Fisioterapia, Recife, PE, Brasil.; 3 Fundação Oswaldo Cruz – Fiocruz, Serviço de Referência Nacional em Filarioses, Centro de Pesquisa Aggeu Magalhães – CPqAM, Recife, PE, Brasil.

**Keywords:** lymphedema, quality of life, physical therapy

## Abstract

**Background:**

Lymphedema of the lower limbs is a chronic disease caused by damage to the lymphatic system that influences people’s mobility, functionality, and quality of life. Questionnaires and physical test are very practical, easy to apply, and low cost methods that provide important data for evaluation of these patients.

**Objectives:**

To evaluate the influence of unilateral lower limb lymphedema on functionality and quality of life, correlating 3 assessment tools.

**Methods:**

This was a descriptive study investigating 25 patients of both sexes with unilateral lymphedema in a lower limb. Limb volume was assessed using circumferential tape measurements, the Medical Outcomes Study Short Form-36 Health Survey (SF-36) was used to assess quality of life, the Lymphoedema Functioning, Disability and Health Questionnaire for Lower Limb Lymphoedema (Lymph-ICF-LL) was used to assess physical, mental, and social skills related to lymphedema, and the Timed Up and Go (TUG) test was used for functional assessment.

**Results:**

Lymphedema was present throughout the affected lower limb of participants. The domains most affected by lymphedema were physical aspects (25.0 ± 31.4) and emotional aspects (36.0 ± 42.9) from the SF-36 and the mobility domain (6.0 ± 2.6) from the Lymph -ICF-LL. Patients performed the TUG in 9.88 ± 1.98 seconds. The TUG was correlated with the questionnaires and the questionnaires were correlated with each other.

**Conclusions:**

People with unilateral lower limb lymphedema exhibited negative impacts on quality of life and functionality, as evaluated by questionnaires, which were correlated with each other. TUG performance was within normal limits, but results correlated with the questionnaires used.

## INTRODUCTION

Lymphedema is a chronic disease provoked by damage or abnormalities affecting the lymphatic system that cause increased limb volume. It affects around 15% of the global population.^1^^,^ 2 Lower limb (LL) lymphedema is the most prevalent presentation, accounting for 80% of cases.^2^^,^ 3

In addition to limb swelling, other very common complaints among patients with lymphedema are pains, reduced amplitude of movement, infections, and problems with body image.^1^^,^ 4 These clinical manifestations determine the impact on patients’ lives and are frequently associated with comorbidities^5^ and psychiatric disorders.^6^

Since the LL are directly related to functionality and independence, lymphedema can impact on aspects such as mobility, functionality, daily activities, professional activities, and social interaction,^2^^,^ 7 ^-^ 9 thereby compromising these patients’ quality of life.^5^^,^ 10 ^-^ 14

Studies evaluating functionality and quality of life in lymphedema do so using assessment methods such as questionnaires, whether disease-specific or generic, and also using physical tests to identify and quantify issues related to the disease and its effects on the different domains of patients’ lives.^11^^,^ 12 ^,^ 15 ^-^ 18

A review study conducted in 2013^18^ with the objective of identifying questionnaires focused on lymphedema related the concepts they covered to concepts from the International Classification of Functioning, Disability and Health (ICF). The results showed that several different questionnaires are used to assess a range of aspects among patients with lymphedema. The Medical Outcomes Study Short Form-36 Health Survey (SF-36) was used by the largest number of studies included in the review. The SF-36 is a generic quality of life questionnaire that has been translated and validated for the Brazilian population. Twelve of the questionnaires identified by the review were specifically for lymphedema. Just one of these questionnaires had a supplement specifically for LL lymphedema and was provided by the authors of the original study to the authors of the review. However, the supplement has not been validated for Brazil.

In 2014, another questionnaire was created specifically for LL lymphedema, the objective of which is to assess the condition’s influence on physical and mental functions and limitations to daily and social activities.^19^ It is entitled the Lymphoedema Functioning, Disability and Health Questionnaire for Lower Limb Lymphoedema (Lymph-ICF-LL) and was translated and culturally adapted for Brazil in 2016.^16^

In addition to questionnaires, physical functionality tests are also used to assess this population, such as the Timed Up And Go (TUG) test. While this test has been validated in Brazil, it is not specific to patients with lymphedema.^5^

Since questionnaires and physical tests are highly practical methods, easy to administer, inexpensive, and provide important data for assessments, and given the important gap with relation to LL lymphedema, this study was conducted with the objective of evaluating the influence of unilateral lower limb lymphedema on functionality and quality of life, correlating the results of three assessment tools.

We stress that this study is a pioneer in using the LL lymphedema-specific questionnaire Lymph-ICF-LL in a Brazilian population, covering several different spheres of life and providing wide-ranging knowledge about the disease and its consequences.

## METHODS

This was a descriptive study conducted between October 2016 and January 2017. The study was approved by the Research Ethics Committee under protocol 1.759.097.

### Population and sample

The study population comprised patients with unilateral lymphedema of a lower limb who were in treatment at or registered with public tertiary centers in the city of Recife, PE, Brazil. The sample size calculation was based on a variable total time to complete the TUG test from a pilot study with 10 patients, a 95% confidence level, and a 10% error, resulting in an estimated sample size of 15 people. All patients eligible for the study were enrolled and assessed, resulting in a final sample size of 25.

Patients of both sexes over the age of 18 were enrolled if they had unilateral lymphedema of an LL categorized as grade I, II, III or IV^20^ and were able to walk independently.

Patients were excluded from the study if they had neurological disorders and/or traumatic orthopedic injuries compromising walking and/or equilibrium, plantar injuries involving the limb with lymphedema, or amputation of the contralateral lower limb, or were illiterate.

### Assessment

After the research objectives and the procedures involved had been explained to them, volunteers signed free and informed consent forms and underwent a physiotherapy assessment including an interview with history taking, LL circumferential measurements, administration of the SF-36 and Lymph-ICF-LL questionnaires, and the TUG test.

General data were collected on patient identification, lymphedema grades,^20^ medications, symptoms, personal and family histories, comorbidities, physiotherapy, and vital signs.

During the physical assessment, each LL was measured at nine different points, taking the apex of the patella as reference (point zero). Four measurements were taken at seven cm intervals above this bony prominence and four measurements were taken below it.^5^

The validated Portuguese translation of the SF-36 was used to assess study participants’ quality of life.^21^ The SF-36 questionnaire comprises 36 questions, grouped in eight domains, as follows: physical functioning, role physical, bodily pain, general health, vitality, social functioning, role emotional, and mental health. It provides an overall score that varies from 0 to 100, where 0 corresponds to worst health status and 100 to best health status.^22^

The Lymph-ICF-LL scale was used to assess lymphedema-related physical, mental, and social abilities. It contains 28 questions, distributed across five domains: physical function, mental function, general tasks/household activities, mobility activities, and life domains/social life. Each question is scored on a numerical scale from 0 to 10, where 0 corresponds to no changes caused by lymphedema and 10 to major health consequences because of the disease.^16^

The TUG test, also known as the stand and walk test,^23^ was used to assess LL functionality, measured in terms of the time taken to complete the test. The subject is instructed to rise from a standardized chair and, on a verbal command, walk 3 meters, turn, walk back to the chair, and sit down. The stop watch is started at the first forward movement of the trunk and stopped when the subject sits back down in the chair, resting their back against it. The test was conducted on a flat surface and patients were requested to walk at a quick, comfortable, and safe pace and were not given any kind of physical assistance.^23^

### Statistical analysis

The data collected were analyzed in an electronic spreadsheet using the Statistical Package for Social Science (SPSS), Chicago, IL, United States, version 20.0.

The results of descriptive analysis for the following sample characterization variables were expressed as absolute and relative frequencies and tabulated: age, sex, physiotherapy regime, limb with lymphedema, lymphedema grade, time since onset of lymphedema, and associated comorbidities. Means and standard deviations were calculated for domain scores from the Lymph-ICF-LL and SF-36 questionnaires and time taken to complete the TUG.

The Shapiro-Wilk test was used to verify normality of variables. Mean values of the circumferential measurements were compared for involved and contralateral limbs using the paired *t* test for parametric variables and the Wilcoxon test for nonparametric variables. Pearson correlation coefficients were calculated for parametric data and Spearman coefficients were used for nonparametric variables. The significance level adopted for this study was p < 0.05.

## RESULTS

**Figure 1:**
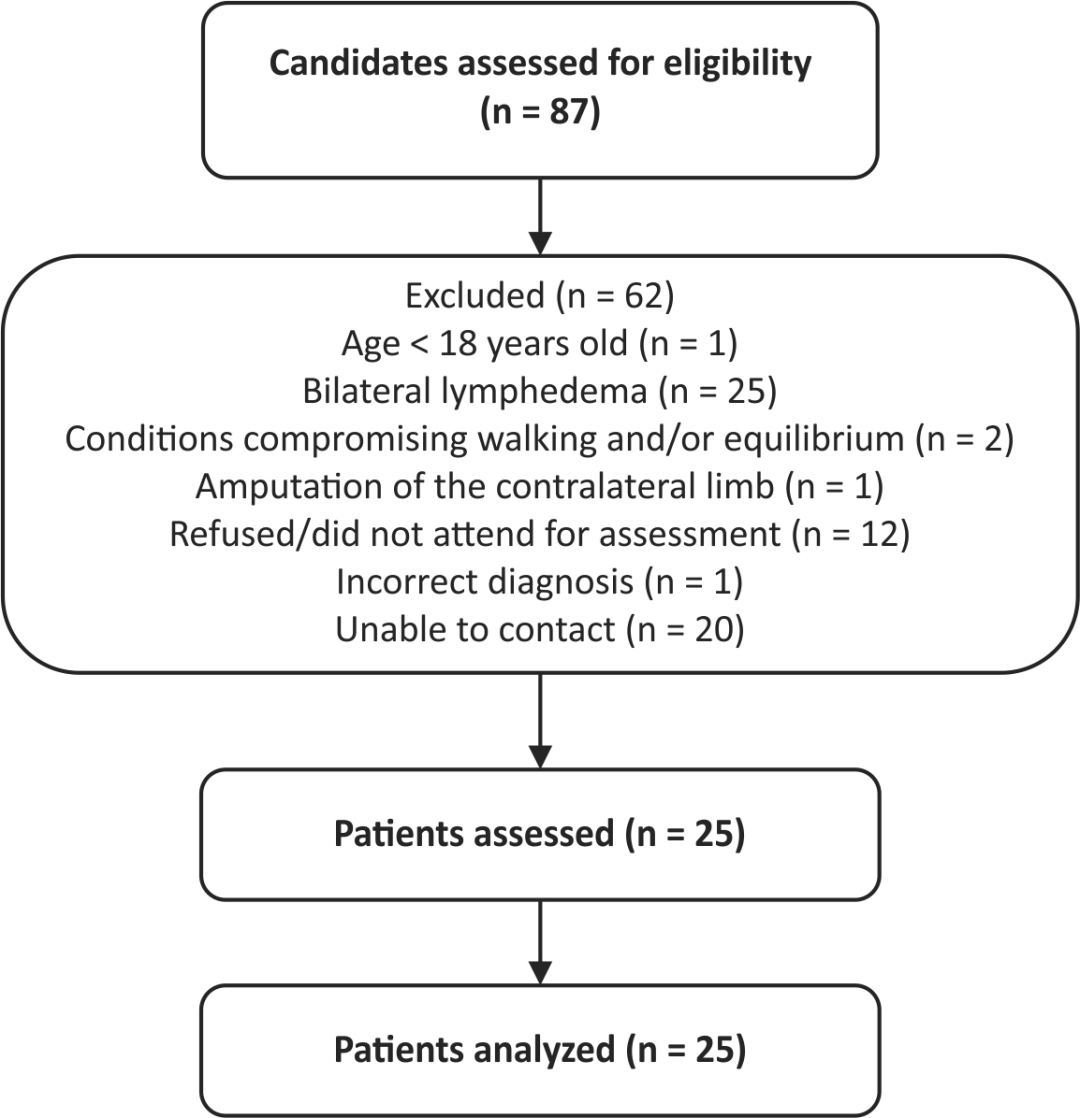
Participant selection flow diagram.

A total of 25 patients were enrolled, as illustrated in Figure 1. Table 1 lists the participants’ sociodemographic and clinical characteristics. The mean age of the sample was 52 years, a majority (72%) were women, lymphedema involving the left lower limb was more frequent (66.7%), and mean body mass index was 35.5 ± 7.4 kg/m^2^.

**Table 1:** Sociodemographic and clinical characteristics of the sample (n = 25).

**Variables**	**n (%)**
Age	
19-39	3 (12%)
40-59	16 (64%)
≥ 60	6 (24%)
Sex	
Male	7 (28%)
Female	18 (72%)
Physiotherapy	
Yes	7 (28%)
No	18 (72%)
Limb involved	
Right lower limb	10 (40%)
Left lower limb	15 (60%)
Lymphedema grade	
I	3 (12%)
II	10 (40%)
III	9 (36%)
IV	3 (12%)
Lymphedema onset	
2 to 5 years	8 (32%)
6 to 10 years	2 (8%)
> 20 years	15 (60%)
Comorbidities and associated conditions	
Smoking	1 (4%)
Alcoholism	10 (40%)
Diabetes	5 (20%)
Hypertension	16 (64%)
Obesity	8 (32%)
Inactivity	19 (76%)

With relation to circumferential measurement results, there were significant differences between the circumferences of the involved and contralateral lower limbs at all nine points measured. This shows that lymphedema was present along the entire length of participants’ involved lower limbs, as shown in Table 2.

**Table 2:** Comparisons between circumferences of involved and contralateral lower limbs of study participants (n = 25).

**Reference points**	**Involved limb** **Mean (± SD)**	**Contralateral limb** **Mean (± SD)**	**p**
+ 28 cm (above)	67.48 (± 9.77)	63.62 (± 7.17)	0.002^*^
+ 21 cm (above)	61.74 (± 9.73)	57.28 (± 6.58)	0.006*
+ 14 cm (above)	56.02 (± 9.97)	50.92 (± 6.40)	0.000^**^
+ 7 cm (above)	51.00 (± 10.74)	45.24 (± 5.29)	0.000**
0 (apex of patella)	45.72 (± 10.48)	39.94 (± 3.99)	0.000**
- 7 cm (below)	48.74 (± 11.96)	40.10 (± 4.65)	0.000**
- 14 cm (below)	48.98 (± 11.92)	38.20 (± 4.67)	0.000**
- 21 cm (below)	42.42 (± 10.63)	31.06 (± 4.83)	0.000**
- 28 cm (ankle)	35.54 (± 8.89)	25.20 (± 4.03)	0.000**

* Paired *t* test;

** Wilcoxon test.

Table 3 lists the domain scores for the SF-36 quality of life questionnaire. Role physical (25.0 ± 31.4), role emotional (36.0 ± 42.9), and functional capacity (45.4 ± 25.9) were the most compromised domains. The study sample had a mean overall SF-36 score of 49.2 ± 22.49.

**Table 3:** Domain scores for The Medical Outcomes Study Short Form-36 Health Survey (SF-36) and the Lymphoedema Functioning, Disability and Health Questionnaire for Lower Limb Lymphoedema (Lymph-ICF-LL) in patients with unilateral lower limb lymphedema (n = 25).

**SF-36 domains**	**Mean ± SD**	**Lymph-ICF-LL domains**	**Mean ± SD**
Physical functioning,	45.4 ± 25.9	Physical function	4.4 ± 1.9
Role physical	25.0 ± 31.4	Mental function	5.6 ± 2.5
Bodily pain,	59.4 ± 35.7	General tasks/household activities	4.8 ± 3.3
General health,	55.1 ± 26.7	Mobility	6.0 ± 2.6
Vitality	46.2 ± 27.2	Life domains/social life	3.9 ± 2.4
Social functioning	66.1 ± 29.6		
Role emotional	36.0 ± 42.9		
Mental health	60.4 ± 24.9		

Table 3 also lists the Lymph-ICF-LL domain scores. Mobility (6.0 ± 2.6) and mental function (5.6 ± 2.5) were the most compromised domains in the patients assessed and life domains/social life (3.9 ± 2.4) was the least affected.

Mean TUG time was 9.88 ± 1.98 s, which is considered satisfactory. This value is based on a study that found that independent adults with no balance problems were able to complete the TUG within 10 s.^24^

Coefficients were calculated for correlations between time taken to complete the TUG and total SF-36 score and Lymph-ICF-LL domain scores, as shown in Table 4. A moderate negative correlation was detected between TUG and total SF-36 score (p = 0.002) and there were moderate positive correlations between TUG and four Lymph-ICF-LL domains (p < 0.01). Lymph-ICF-LL life domains/social life had a weak and non-significant correlation (p = 0.713) with TUG time.

**Table 4:** Correlation between total TUG time, overall SF-36 score and Lymph-ICF-LL domain scores.

**Variables**	**Correlation coefficient**	**p**
TUG vs. Overall score (SF-36)^P^	-0.584^**^	0.002
TUG vs. Physical function (Lymph-ICF-LL)^S^	0.685**	0.000
TUG vs. Mental function (Lymph-ICF-LL)^S^	0.522**	0.007
TUG vs. General tasks/household activities (Lymph-ICF-LL)^S^	0.519**	0.008
TUG vs. Mobility (Lymph-ICF-LL)^P^	0.584**	0.002
TUG vs. Life domains/social life (Lymph-ICF-LL)^P^	0.077	0.713

TUG: Total time to complete the Timed Up and Go test; SF-36: The Medical Outcomes Study Short Form-36 Health Survey; Lymph-ICF-LL: Lymphoedema Functioning, Disability and Health Questionnaire for Lower Limb Lymphoedema; ^P^Pearson correlation; ^S^Spearman correlation;

** p < 0.01.

Correlations between Lymph-ICF-LL domains and SF-36 domains (Table 5) were negative and high or moderate (p < 0.01). The strongest correlations were between the Lymph-ICF-LL mobility domain and the SF-36 functional capacity domain (p = 0.000) and between the Lymph-ICF-LL life domains/social life domain and the SF-36 social functioning domain (p = 0.000).

**Table 5:** Correlations between Lymph-ICF-LL domains with and SF-36 domains.

**Variables**	**Correlation coefficient**	**p**
Physical function (Lymph-ICF-LL) vs. Functional capacity (SF-36)^S^	-0.665^**^	0.000
Mental function (Lymph-ICF-LL) vs. Mental health (SF-36)^S^	-0.508**	0.010
General tasks/household activities (Lymph-ICF-LL) vs. General health (SF-36)^S^	-0.564**	0.003
Mobility (Lymph-ICF-LL) vs. Functional capacity (SF-36)^P^	-0.814**	0.000
Life domains/social life (Lymph-ICF-LL) vs. Role social (SF-36)^S^	-0.748**	0.000

SF-36: The Medical Outcomes Study Short Form-36 Health Survey; Lymph-ICF-LL: Lymphoedema Functioning, Disability and Health Questionnaire for Lower Limb Lymphoedema; ^P^Pearson correlation; ^S^Spearman correlation;

** p < 0.01.

## DISCUSSION

It is known that lymphedema affects people of a range of different ages and of both sexes. It primarily occurs among women,^2^ as confirmed in this study, in which the sample was predominantly female (72%). This is similar to two other studies, in which samples comprised 70.7% and 77% women.^11^^,^ 12 In the present study, women appeared more accessible and more attuned to the study objectives, in addition to being more concerned with health issues.

The majority of the participants in this study were not receiving physiotherapy, even though it is known that physiotherapy is part of the gold standard treatment for these patients.^1^^,^ 25 Additionally, the majority of them had been living with lymphedema for more than 20 years, confirming the chronic nature of the disease. In view of this, we stress the importance of controlling the chronic condition with appropriate treatment, which can make a positive contribution to quality of life in this population.^5^

These patients had associated comorbidities, in line with the literature,^5^^,^ 26 ^,^ 27 confirming that they are common in people with severe lymphatic dysfunction.^28^ Arterial hypertension, obesity, and diabetes were the most common comorbidities, as in a study by Santana et al.,^26^ who state that if these conditions are not controlled they facilitate development of sequelae. Soares et al.^5^ reported these three comorbidities in a clinical study of patients with lymphedema living in an area where filariasis is endemic. In addition to these conditions, inactivity was also prevalent in the present study and, since it is directly associated with obesity and diabetes,^29^ it is linked with worse lymphedema prognosis.^26^

Irrespective of lymphedema grade, circumferential measurements provide quantitative data that can be used to categorize lymphedema severity. According to the American Physical Therapy Association,^30^ lymphedema is considered moderate when there are differences between limbs in the range of 3 to 5 cm and severe when the difference exceeds 5 cm. In this study, comparison between involved and contralateral limbs revealed significant differences between all LL circumference measurements at all levels. Differences between limbs exceeding 5 cm were observed at the majority of measurement points.

The study also detected compromised health-related quality of life in all SF-36 domains, in comparison with mean normal values for the Brazilian population found in the literature,^31^ demonstrating that the participants’ quality of life was compromised. Our data confirm previous studies,^5^^,^ 8 ^,^ 11 ^,^ 28 ^-^ 30 identifying the domains role physical (25.0 ± 31.4), role emotional (36.0 ± 42.9), and functional capacity (45.4 ± 25.9) as most severely affected.

The increased volume of the affected limb and the extra weight it causes, restricted movements, pain, and episodes of erysipelas in the involved limb were frequent complaints among the patients in this study. According to the participants, these conditions were responsible for triggering psychological problems over time, possibly explaining the SF-36 findings.

Our results partially agree with those of a study conducted with patients with lymphedema enrolled at a wound management and vascular clinic in Ireland. That study also used the SF-36 and observed that functional capacity and role physical were the domains most affected, while role emotional was less compromised.^32^

To our knowledge, this is the first study to administer the Lymph-ICF-LL questionnaire to this population. There are a number of questionnaires specifically for lymphedema available internationally, but to date this is the only one that has been translated and culturally adapted for Brazil.^11^^,^ 16 ^,^ 17

The Lymph-ICF-LL was used to assess physical, mental, and social abilities in relation to lymphedema. It does not classify patients, but provides a score which indicates greater health consequences from lymphedema the closer it approaches to 10. The mobility (6.0 ± 2.6) and mental function (5.6 ± 2.5) domains were scored closest to 10 and, therefore, were the most severely affected.

We believe that the finding with relation to mobility is particularly pertinent, since reduced mobility can be explained by the increased volume of the limb and its consequent extra weight. This makes it more likely that joint movements will be restricted and that the patient will suffer pain, causing overloads that directly impact on their mobility and functionality.^2^^,^ 7 ^,^ 8 ^,^ 9 ^,^ 33

With regard to mental function, the literature states that people with lymphedema suffer from significant psychiatric disorders, such as anxiety and depression, which affect perceptions of body image, interpersonal relations, and sexual relations, and also make daily activities more difficult. The clinical condition also creates a sensation of impotence, fears of incapacity, and problems with interpersonal relations because patients are embarrassed to expose the limb that has lymphedema.^6^

With relation to the TUG test, in our results the total time to complete the test was considered satisfactory (9.88 ± 1.98 seconds), based on a study by Figueiredo et al.^24^ This finding surprises us, since mobility was the most compromised domain according to the Lymph-ICF-LL questionnaire. However, examining the issue in more detail reveals that the items in this questionnaire are more specific and demand greater limb mobility, and also agility, such as in the following items: 18) kneeling; 19) walking (2 kilometers); 20) riding a bicycle; 21) driving a car; and 22) climbing stairs (or boarding and descending from a bus).

Although the TUG is used to evaluate LL functionality and mobility and has been validated for Brazil, it is not an assessment method specifically for people with lymphedema.^5^ Nevertheless, we believe that the values observed fall within the expected range, since it is a test that requires patients to rise, walk, and sit down, and the study participants had chronic lymphedema. Since walking is a movement that we automate, the patients assessed may have adapted to this automation.

Our findings show that there is a correlation between the time taken to complete the TUG test and total SF-36 score and also with Lymph-ICF-LL domains. The analysis of TUG against total SF-36 score revealed a moderate negative correlation, indicating that the longer the subject took to complete the test, the lower the SF-36 score, which means worse quality of life. These data are in line with the literature, which associates poor functionality with low quality of life indexes in this population.^2^^,^ 8 ^,^ 9

Positive correlations were observed between the time taken to complete the TUG test and the Lymph-ICF-LL domains, suggesting that the worse the performance in the test and the slower the subject, the higher the scores in the questionnaire domains and, consequently, the compromise to the functions assessed caused by the lymphedema.

Since two questionnaires were administered, one lymphedema-specific and the other generic, we compared the results for the Lymph-ICF-LL domains with the SF-36 domains that were most compatible, in order to analyze whether they were correlated. We found high and moderate negative correlations, demonstrating the applicability of the Lymph-ICF-LL to this population, even though it has not yet been validated for Brazil. We back up the data in the literature showing that the greater the influence lymphedema has on a patient’s health, the worse quality of life becomes,^7^^,^ 8 since higher scores on the Lymph-ICF-LL were associated with lower SF-36 scores.

In this respect, there is a need for greater focus on these patients’ functionality and quality of life, since both proved to be negatively influenced by the disease. We stress the applicability of assessment instruments such as the Lymph-ICF-LL and SF-36 questionnaires and the TUG test for assessing this population. They correlate with each other and their results can contribute to the scientific literature and to clinical practice, enabling better understanding of prognosis and improved disease management. We also highlight the pioneering nature of this study in using a disease-specific instrument recently made available in Brazil.

## CONCLUSIONS

This study found evidence that people with unilateral lymphedema of a lower limb exhibit a negative impact on quality of life and functionality related physical, mental, and social abilities assessed using questionnaires, which correlate with each other. While the time taken to complete the TUG test was within normal limits, it was also observed that times correlated with the SF-36 and Lymph-ICF-LL questionnaires.

Since this study is the first to administer the Lymph-ICF-LL to this population, additional studies are needed to compare results and enable more robust interpretations of the findings of this study.
