# Supplementary material for: Functionality and quality of life of patients with unilateral lymphedema of a lower limb: a cross-sectional study
Source: J Vasc Bras. 2019 May 7;18:e20180066. doi: 10.1590/1677-5449.006618 (PMC6632670; doi:10.1590/1677-5449.006618)
Supplement: Translation

# Translation: Funcionalidade e qualidade de vida em indivíduos com linfedema unilateral em membro inferior: um estudo transversal

**Authors:** Barbara Cristina de Sousa Pedrosa, Juliana Netto Maia, Ana Paula de Lima Ferreira, Maria das Graças Rodrigues de Araújo, Eduardo José Nepomuceno Montenegro, Fernando Leonel da Silva, Célia Maria Machado Barbosa de Castro, Maria do Amparo Andrade

**Affiliations:** 1 Universidade Federal de Pernambuco – UFPE, Laboratório de Imunopatologia Keizo Asami – LIKA, Recife, PE, Brasil.; 2 Universidade Federal de Pernambuco – UFPE, Departamento de Fisioterapia, Recife, PE, Brasil.; 3 Fundação Oswaldo Cruz – Fiocruz, Serviço de Referência Nacional em Filarioses, Centro de Pesquisa Aggeu Magalhães – CPqAM, Recife, PE, Brasil.

**Keywords:** linfedema, qualidade de vida, fisioterapia

## Abstract

**Contexto:**

O linfedema de membros inferiores é uma doença crônica decorrente de dano no sistema linfático que influencia a mobilidade, a funcionalidade e a qualidade de vida dos indivíduos. Questionários e o teste físico são métodos bastante práticos, de fácil aplicação e baixo custo, que fornecem dados importantes para a avaliação desses pacientes.

**Objetivos:**

Avaliar a influência do linfedema unilateral de membro inferior na funcionalidade e na qualidade de vida, correlacionando três ferramentas de avaliação.

**Métodos:**

Estudo descritivo com 25 indivíduos com linfedema unilateral em membro inferior, de ambos os sexos. Foi avaliada a perimetria e foram aplicados *The Medical Outcome Study Short Form-36 Health Survey* (SF-36) para avaliação da qualidade de vida, *Lymphoedema Functioning, Disability and Health Questionnaire for Lower Limb Lymphoedema* (Lymph-ICF-LL) para estudo das habilidades físicas, mentais e sociais relacionadas ao linfedema e o *Timed Up and Go* (TUG) para avaliação da funcionalidade.

**Resultados:**

Houve a presença de linfedema em todo o membro inferior dos participantes. Os domínios mais prejudicados pelo linfedema foram os aspectos físicos (25,0 ± 31,4) e emocionais (36,0 ± 42,9) no SF-36 e o domínio mobilidade (6,0 ± 2,6) no Lymph-ICF-LL. O TUG foi realizado em 9,88 ± 1,98 s. Houve correlação entre o TUG e os questionários e entre os dois questionários utilizados.

**Conclusões:**

Indivíduos com linfedema unilateral em membro inferior apresentam um impacto negativo na qualidade de vida e na funcionalidade avaliadas através de questionários, que correlacionam entre si. Não foi encontrada alteração no TUG, mas houve correlação entre ele e os questionários utilizados.

## INTRODUÇÃO

O linfedema é uma doença crônica provocada por danos ou anomalias no sistema linfático que promovem o aumento do volume do membro. Acomete cerca de 15% da população mundial^1^^,^ 2. Entre os tipos de linfedema, o de membros inferiores (MMII) tem uma maior prevalência, sendo responsável por 80% dos casos^2^^,^ 3.

Juntamente com volume do membro, outras queixas bastante comuns nos pacientes com linfedema são dores, diminuição da amplitude de movimento, infecções e problemas com a imagem corporal^1^^,^ 4. Essas manifestações clínicas determinam o impacto provocado na vida desses indivíduos e estão frequentemente associadas a comorbidades^5^ e distúrbios psiquiátricos^6^.

Como os MMII estão diretamente relacionados a funcionalidade e independência, o linfedema pode influenciar aspectos como mobilidade, funcionalidade, atividades da vida diária, atividades profissionais e interação social^2^^,^ 7 ^-^ 9. Assim, compromete a qualidade de vida desses pacientes^5^^,^ 10 ^-^ 14.

Os estudos que avaliam funcionalidade e qualidade de vida no linfedema o fazem através de métodos avaliativos como questionários, sejam eles específicos para a doença ou genéricos, e também através de testes físicos a fim de identificar e quantificar questões referentes à doença e sua influência nos diferentes domínios que envolvem a vida dos indivíduos acometidos^11^^,^ 12 ^,^ 15 ^-^ 18.

Um estudo de revisão de 2013^18^ teve como objetivo identificar questionários com foco no linfedema, relacionando conceitos presentes neles aos conceitos da Classificação Internacional de Funcionalidade, Incapacidade e Saúde (CIF). Os resultados apontam vários questionários que são utilizados para avaliar diversos aspectos das pessoas com linfedema. O *The Medical Outcome Study Short Form-36 Health Survey* (SF-36), que é um questionário genérico de qualidade de vida, foi o mais utilizado pelos estudos incluídos na revisão e constitui um questionário validado na população brasileira. Além desse, 12 dos questionários encontrados pela revisão eram específicos para o linfedema. Contudo, apenas um questionário envolvia um suplemento específico para o linfedema de MMII, que foi disponibilizado pelos autores do estudo original aos autores da revisão. Porém, tal suplemento não é validado no Brasil.

Em 2014, foi criado outro questionário específico para o linfedema de MMII, cujo objetivo é avaliar sua influência nas funções física e mental, limitações na realização de atividades e vida social^19^. Trata-se do *Lymphoedema Functioning, Disability and Health Questionnaire for Lower Limb Lymphoedema* (Lymph-ICF-LL), que foi traduzido e adaptado transculturalmente para o Brasil em 2016^16^.

Além dos questionários, testes físicos de funcionalidade também vêm sendo empregados para a avaliação dessa população, como o *Timed Up And Go* (TUG). Apesar de ser validado no Brasil, ele não é específico para indivíduos com linfedema^5^.

Sendo os questionários e o teste físico métodos bastante práticos, de fácil aplicação e baixo custo, que fornecem dados importantes para a avaliação, e diante da importante lacuna envolvendo o linfedema de MMII, este trabalho teve como objetivo avaliar a influência do linfedema unilateral de membro inferior na funcionalidade e na qualidade de vida, correlacionando três ferramentas de avaliação.

Ressaltamos que este estudo surge como pioneiro na utilização do questionário específico para linfedema de MMII Lymph-ICF-LL na população brasileira, abordando os diversos âmbitos da vida e fornecendo um amplo conhecimento acerca da doença e seus acometimentos.

## MÉTODOS

Trata-se de um estudo do tipo descritivo, desenvolvido no período de outubro de 2016 a janeiro de 2017. O estudo foi aprovado pelo Comitê de Ética, sob o número de parecer 1.759.097.

### População e amostra

A população do estudo foi composta por indivíduos com linfedema unilateral em membro inferior, em acompanhamento e/ou cadastrados em serviços públicos de referência para a doença na cidade do Recife, PE. Adotou-se o cálculo amostral para a variável tempo total para a realização do teste TUG a partir de um piloto com 10 pacientes, utilizando-se um nível de confiança de 95% e um erro de 10%, chegando-se a 15 indivíduos. Todos os pacientes elegíveis para o estudo foram avaliados e a amostra foi composta por 25 indivíduos.

Foram incluídos indivíduos de ambos os sexos, com idade superior a 18 anos, portadores de linfedema unilateral em um dos MMII categorizados nos graus I, II, III ou IV^20^ e que apresentavam marcha independente.

Foram excluídos do estudo aqueles que apresentaram desordens neurológicas e/ou comprometimento traumato-ortopédico que prejudicasse marcha e/ou equilíbrio, lesões em região plantar no membro acometido pelo linfedema, amputação em membro inferior contralateral e indivíduos não alfabetizados.

### Avaliação

Após serem esclarecidos sobre os objetivos da pesquisa e os devidos procedimentos, os voluntários assinaram o termo de consentimento livre e esclarecido e foram submetidos a uma avaliação fisioterapêutica que incluiu entrevista com anamnese, aferição da perimetria dos MMII, utilização dos questionários SF-36 e Lymph-ICF-LL e desempenho no teste TUG.

Foram registradas informações gerais sobre identificação, graus do linfedema^20^, medicação em uso, sintomas, antecedentes pessoais e familiares, comorbidades, realização de fisioterapia e sinais vitais dos participantes.

Na perimetria, foram medidos nove pontos de cada um dos MMII, tomando-se como referência o ápice da patela (ponto zero). Foram realizadas quatro medidas a cada sete centímetros acima do acidente ósseo e quatro medidas abaixo^5^.

O SF-36 foi utilizado para a avaliação da qualidade de vida dos participantes do estudo. Foi utilizada sua versão validada e traduzida para a língua portuguesa^21^. Esse questionário é composto por 36 questões, agrupadas em oito domínios: capacidade funcional, aspectos físicos, dor, estado geral de saúde, vitalidade, aspectos sociais, aspectos emocionais e saúde mental. Possui um escore final que varia de 0 a 100, em que 0 corresponde ao pior estado geral de saúde e 100 ao melhor estado de saúde^22^.

O Lymph-ICF-LL foi utilizado para avaliar as habilidades físicas, mentais e sociais relacionadas ao linfedema. Ele compreende 28 questões, que são distribuídas em cinco domínios: função física, função mental, atividades gerais/domiciliares, mobilidade e domínios da vida/vida social. Para cada questão, a pontuação a ser marcada varia em uma escala numérica de 0 a 10, em que 0 corresponde a nenhuma alteração decorrente do linfedema e 10 a grandes consequências para a saúde devido à doença^16^.

O TUG, também conhecido como teste de levantar e andar^23^, foi empregado para avaliação da funcionalidade dos MMII, medido através do tempo de realização do teste. Para a realização do teste, o indivíduo foi orientado a levantar-se de uma cadeira padronizada e, após o comando verbal, andar 3 metros, se virar, andar de volta à cadeira e se sentar. O cronômetro era disparado ao primeiro movimento anterior do tronco e cessado quando o indivíduo sentava na cadeira e apoiava as costas. O teste foi realizado em superfície regular e plana, e os pacientes foram instruídos a andar em ritmo rápido, confortável e seguro, sem receber qualquer assistência física^23^.

### Análise estatística

Os dados obtidos foram analisados em planilha eletrônica através do pacote estatístico *Statistical Package for Social Science* (*SPSS*), Chicago, IL, EUA, versão 20.0.

A análise descritiva foi apresentada em tabelas através de frequências absolutas e relativas para as seguintes variáveis de caracterização da amostra: idade, sexo, realização de fisioterapia, membro acometido pelo linfedema, grau do linfedema, tempo de início do linfedema e comorbidades associadas. Foram utilizados médias e desvios padrão para os domínios do questionário Lymph-ICF-LL, do SF-36 e tempo de realização do TUG.

Para testar a normalidade das variáveis, foi realizado o teste de Shapiro-Wilk. Para comparar os valores médios da perimetria entre os membros acometidos e não acometidos, foram utilizados o teste T pareado para os dados paramétricos e o teste de Wilcoxon para os não paramétricos. Foram utilizadas a correlação de Pearson para dados paramétricos e a correlação de Spearman para os não paramétricos. O nível de significância adotado no estudo foi p < 0,05.

## RESULTADOS

Foram avaliados 25 pacientes, como demonstra a Figura 1. As características sociodemográficas e clínicas desses indivíduos encontram-se descritas na Tabela 1. A amostra apresentou uma média de 52 anos de idade e foi composta, principalmente, por mulheres (72%), com predomínio do linfedema no membro inferior esquerdo (66,7%) e valor médio de índice de massa corporal de 35,5 ± 7,4 kg/m^2^.

**Figura 1:** Fluxograma de seleção dos participantes.

**Tabela 1:** Caracterização da amostra quanto às características sociodemográficas e clínicas (n = 25).

**Variáveis**	**n (%)**
Idade	
19-39	3 (12%)
40-59	16 (64%)
≥ 60	6 (24%)
Sexo	
Masculino	7 (28%)
Feminino	18 (72%)
Fisioterapia	
Sim	7 (28%)
Não	18 (72%)
Membro acometido	
Membro inferior direito	10 (40%)
Membro inferior esquerdo	15 (60%)
Grau do linfedema	
I	3 (12%)
II	10 (40%)
III	9 (36%)
IV	3 (12%)
Início do linfedema	
2 a 5 anos	8 (32%)
6 a 10 anos	2 (8%)
> 20 anos	15 (60%)
Comorbidades e condições associadas	
Fumo	1 (4%)
Etilismo	10 (40%)
Diabetes	5 (20%)
Hipertensão	16 (64%)
Obesidade	8 (32%)
Sedentarismo	19 (76%)

Com relação à perimetria, houve diferenças significativas entre as circunferências do membro inferior acometido e do não acometido em todos os nove pontos mensurados. Esse dado demonstra que o linfedema estava presente ao longo de todo o membro inferior dos participantes, conforme mostra a Tabela 2.

**Tabela 2:** Comparação entre a circunferência dos membros inferiores acometidos e não acometidos pelo linfedema nos participantes do estudo (n = 25).

**Pontos de referência**	**Membro acometido** **Média (± DP)**	**Membro não acometido** **Média (± DP)**	**Valor de p**
+ 28 cm (acima)	67,48 (± 9,77)	63,62 (± 7,17)	0,002^*^
+ 21 cm (acima)	61,74 (± 9,73)	57,28 (± 6,58)	0,006*
+ 14 cm (acima)	56,02 (± 9,97)	50,92 (± 6,40)	0,000^**^
+ 7 cm (acima)	51,00 (± 10,74)	45,24 (± 5,29)	0,000**
0 (ápice da patela)	45,72 (± 10,48)	39,94 (± 3,99)	0,000**
- 7 cm (abaixo)	48,74 (± 11,96)	40,10 (± 4,65)	0,000**
- 14 cm (abaixo)	48,98 (± 11,92)	38,20 (± 4,67)	0,000**
- 21 cm (abaixo)	42,42 (± 10,63)	31,06 (± 4,83)	0,000**
- 28 cm (tornozelo)	35,54 (± 8,89)	25,20 (± 4,03)	0,000**

* Teste T pareado;

** Teste de Wilcoxon.

Na Tabela 3, estão apresentados os valores dos domínios do questionário de qualidade de vida SF-36. Aspectos físicos (25,0 ± 31,4), aspectos emocionais (36,0 ± 42,9) e capacidade funcional (45,4 ± 25,9) foram os domínios mais prejudicados. O escore final do SF-36 dos participantes do estudo apresentou uma média de 49,2 ± 22,49.

**Tabela 3:** Escore por domínios do *The Medical Outcome Study Short Form-36 Health Survey* (SF-36) e do *Lymphoedema Functioning, Disability and Health Questionnaire for Lower Limb Lymphoedema* (Lymph-ICF-LL) em indivíduos com linfedema unilateral em membro inferior (n = 25).

**Domínios do questionário SF-36**	**Média ± DP**	**Domínios do questionário Lymph-ICF-LL**	**Média ± DP**
Capacidade funcional	45,4 ± 25,9	Função física	4,4 ± 1,9
Aspectos físicos	25,0 ± 31,4	Função mental	5,6 ± 2,5
Dor	59,4 ± 35,7	Atividades gerais/domiciliares	4,8 ± 3,3
Estado geral de saúde	55,1 ± 26,7	Mobilidade	6,0 ± 2,6
Vitalidade	46,2 ± 27,2	Domínios da vida social	3,9 ± 2,4
Aspectos sociais	66,1 ± 29,6		
Aspectos emocionais	36,0 ± 42,9		
Saúde mental	60,4 ± 24,9		

Ainda na Tabela 3, estão apresentados os domínios do Lymph-ICF-LL. A mobilidade (6,0 ± 2,6) e a função mental (5,6 ± 2,5) foram os domínios que mais apresentaram alterações nos pacientes avaliados, e os domínios da vida/vida social (3,9 ± 2,4) foram os menos alterados.

Com relação ao TUG, o tempo de realização do teste foi de 9,88 ± 1,98 s, sendo considerado satisfatório. Esse valor tem base em estudo que constatou que indivíduos adultos, independentes e sem alterações no equilíbrio são capazes de realizar o TUG em até 10 s^24^.

Foram realizadas correlações do tempo de realização do TUG com o escore final do SF-36 e com os domínios do Lymph-ICF-LL, conforme detalha a Tabela 4. Foi encontrada uma correlação moderada negativa entre o TUG e o escore final do SF-36 (p = 0,002) e correlações moderadas positivas entre o TUG e quatro domínios do Lymph-ICF-LL (p < 0,01). Apenas os domínios da vida social do Lymph-ICF-LL apresentaram correlação fraca e não significativa (p = 0,713).

**Tabela 4:** Correlação do tempo total de realização do TUG com o escore final do SF-36 e com os domínios do Lymph-ICF-LL.

**Variáveis**	**Coeficiente de correlação**	**Valor de p**
TUG *vs.* Escore final (SF-36)^P^	-0,584^**^	0,002
TUG *vs.* Função física (Lymph-ICF-LL)^S^	0,685**	0,000
TUG *vs.* Função mental (Lymph-ICF-LL)^S^	0,522**	0,007
TUG *vs.* Atividades gerais/domiciliares (Lymph-ICF-LL)^S^	0,519**	0,008
TUG *vs.* Mobilidade (Lymph-ICF-LL)^P^	0,584**	0,002
TUG *vs.* Domínios da vida social (Lymph-ICF-LL)^P^	0,077	0,713

TUG: Tempo total no *Timed Up and Go*; SF-36: *The Medical Outcome Study Short Form-36 Health Survey*; Lymph-ICF-LL: *Lymphoedema Functioning, Disability and Health Questionnaire for Lower Limb Lymphoedema*; ^P^Correlação de Pearson; ^S^Correlação de Spearman;

** Valor de p < 0,01.

Ao se correlacionar os domínios do Lymph-ICF-LL com domínios do SF-36 (Tabela 5), observaram-se correlações negativas altas e moderadas (p < 0,01), com destaque para as correlações mais fortes que ocorreram entre o domínio mobilidade do Lymph-ICF-LL e o domínio capacidade funcional do SF-36 (p = 0,000) e entre os domínios da vida social do Lymph-ICF-LL e o domínio aspectos sociais do SF-36 (p = 0,000).

**Tabela 5:** Correlação dos domínios do Lymph-ICF-LL com os domínios do SF-36.

**Variáveis**	**Coeficiente de correlação**	**Valor de p**
Função física (Lymph-ICF-LL) *vs.* Capacidade funcional (SF-36)^S^	-0,665^**^	0,000
Função mental (Lymph-ICF-LL) *vs.* Saúde mental (SF-36)^S^	-0,508**	0,010
Atividades gerais/domiciliares (Lymph-ICF-LL) *vs.* Estado geral de saúde (SF-36)^S^	-0,564**	0,003
Mobilidade (Lymph-ICF-LL) *vs.* Capacidade funcional (SF-36)^P^	-0,814**	0,000
Domínios da vida social (Lymph-ICF-LL) *vs.* Aspectos sociais (SF-36)^S^	-0,748**	0,000

SF-36: *The Medical Outcome Study Short Form-36 Health Survey*; Lymph-ICF-LL: *Lymphoedema Functioning, Disability and Health Questionnaire for Lower Limb Lymphoedema*; ^P^Correlação de Pearson; ^S^Correlação de Spearman;

** Valor de p < 0,01.

## DISCUSSÃO

Sabe-se que o linfedema acomete indivíduos em diferentes faixas etárias e de ambos os sexos. Ocorre principalmente entre as mulheres^2^, achado confirmado neste estudo, que apresentou uma amostra composta predominantemente pelo sexo feminino (72%). Esse resultado foi semelhante aos de dois estudos que obtiveram amostras compostas por 70,7% e 77% de mulheres^11^^,^ 12. No presente estudo, as mulheres se mostraram mais acessíveis e sensibilizadas aos objetivos do estudo, além de se mostrarem mais preocupadas com questões de saúde.

A maioria dos participantes deste estudo não realizava fisioterapia, embora saiba-se que ela está incluída no tratamento considerado padrão-ouro para esses pacientes^1^^,^ 25. Além disso, a maioria deles convive com o linfedema há mais de 20 anos, o que corrobora o caráter crônico da doença. Diante disso, reforçamos a importância do controle do agravo crônico através do tratamento adequado, que pode contribuir positivamente para a qualidade de vida dessa população^5^.

Os indivíduos apresentaram comorbidades associadas, o que está em conformidade com a literatura^5^^,^ 26 ^,^ 27 e confirma que elas são comuns em pessoas com disfunção linfática grave^28^. A hipertensão arterial, a obesidade e o diabetes foram as principais comorbidades encontradas, tal qual no estudo de Santana et al.^26^, que defende que tais agravos, se não forem controlados, favorecem a evolução de sequelas. Soares et al.^5^ reporta a existência das três comorbidades em seu ensaio clínico envolvendo pacientes com linfedema residentes em área endêmica de filariose. Além dessas, o sedentarismo teve destaque no presente estudo e, estando diretamente associado à obesidade e à ocorrência de diabetes^29^, leva ao agravamento do prognóstico do linfedema^26^.

Independentemente do grau do linfedema, a perimetria fornece dados quantitativos que possibilitam categorizar a severidade do linfedema. Segundo a *American Physical Therapy Association* 30, o linfedema é considerado moderado quando há diferenças de 3 a 5 cm entre os membros aferidos e severo quando essa diferença for superior a 5 cm. Neste estudo, na comparação entre membro acometido e não acometido, houve diferenças significativas entre todas as medições da circunferência dos MMII dos participantes do estudo. Diferenças intermembros maiores que 5 cm foram encontradas na maioria dos pontos aferidos do membro inferior dos participantes.

O presente estudo também demonstrou um comprometimento da qualidade de vida relacionada à saúde em todos os domínios do SF-36 em comparação aos valores normativos médios da população brasileira encontrados na literatura^31^, evidenciando um prejuízo na qualidade de vida desses participantes. Nossos dados ratificam estudos anteriores^5^^,^ 8 ^,^ 11 ^,^ 28 ^-^ 30 e apontam os domínios aspectos físicos (25,0 ± 31,4), aspectos emocionais (36,0 ± 42,9) e capacidade funcional (45,4 ± 25,9) como os mais comprometidos.

O volume do membro acometido e consequente aumento do seu peso, a presença de movimentos reduzidos, dores e episódios de erisipela no membro acometido foram queixas frequentemente relatadas pelos pacientes nesta pesquisa. Segundo os participantes, esses foram responsáveis por desencadear acometimentos psicológicos ao longo do tempo, constituindo possíveis explicações para os achados do SF-36.

Nossos resultados corroboram parcialmente os de um estudo desenvolvido com pacientes com linfedema provenientes de serviços de tratamento vascular e de feridas na Irlanda. Foi observado também, com a utilização do SF-36, que a capacidade funcional e a limitação por aspectos físicos foram os domínios mais prejudicados, enquanto a limitação por aspectos emocionais foi a menos prejudicada^32^.

De nosso conhecimento, este estudo é pioneiro na utilização do questionário Lymph-ICF-LL nessa população. Existem alguns questionários específicos para linfedema disponíveis internacionalmente, mas, no Brasil, apenas esse foi traduzido e adaptado transculturalmente até o presente momento^11^^,^ 16 ^,^ 17.

O Lymph-ICF-LL foi empregado para observação das habilidades físicas, mentais e sociais relacionadas ao linfedema. Ele não classifica os indivíduos avaliados, apenas oferece um escore que, quanto mais próximo a 10, mais sugere consequências do linfedema para a saúde. Os domínios mobilidade (6,0 ± 2,6) e função mental (5,6 ± 2,5) foram aqueles que apresentaram valores mais próximos a 10, sendo, portanto, os mais prejudicados.

Acreditamos que o achado referente à mobilidade seja pertinente, uma vez que a redução da mobilidade pode ser justificada pelo aumento do volume do membro e, consequentemente, do seu peso. Isso favorece o surgimento da limitação dos movimentos articulares e de dor, impondo sobrecargas que influenciam diretamente a mobilidade e a funcionalidade desses indivíduos^2^^,^ 7 ^,^ 8 ^,^ 9 ^,^ 33.

Quanto à função mental, a literatura aponta que pessoas com linfedema apresentam distúrbios psiquiátricos importantes como ansiedade e depressão, que afetam a percepção da imagem corporal, as relações interpessoais e as relações sexuais, dificultando também as atividades de vida diária. A condição clínica promove ainda uma sensação de impotência, de medo das incapacidades, bem como dificuldades nas relações interpessoais devido ao sentimento de vergonha ao expor o membro com linfedema^6^.

Com relação ao TUG, diante dos nossos resultados, o tempo total para realização do teste foi considerado satisfatório (9,88 ± 1,98 segundos), com base no estudo de Figueiredo et al.^24^. Esse achado nos surpreende, uma vez que o domínio mobilidade do questionário Lymph-ICF-LL foi o mais prejudicado pelo linfedema. Porém, ao aprofundarmos a questão, as perguntas do questionário são mais específicas e exigem uma maior mobilidade do membro, bem como agilidade, como nas seguintes questões: 18) Ajoelhar-se; 19) Caminhar (2 quilômetros); 20) Andar de bicicleta; 21) Dirigir um carro; e 22) Subir escadas (ou subir e descer do ônibus).

Embora o TUG seja utilizado para avaliação da funcionalidade e mobilidade funcional de MMII e ser validado no Brasil, não se trata de um método avaliativo específico para indivíduos com linfedema^5^. Ainda assim, acreditamos que pode ter apresentado valores dentro do esperado, uma vez que se trata de um teste que exige levantar, andar e sentar, e os indivíduos do estudo apresentam linfedema crônico. Como a marcha é um movimento que automatizamos, os pacientes avaliados podem já estar adaptados a essa automatização.

Nossos achados mostram que existe uma correlação do tempo de realização do TUG com o escore final do SF-36 e com os domínios do Lymph-ICF-LL. Ao correlacionar o TUG com o escore final do SF-36, foi encontrada uma correlação moderada negativa, evidenciando que quanto maior o tempo gasto pelo indivíduo para realizar o teste, menor sua pontuação no SF-36, que significa pior qualidade de vida. Esses dados estão em conformidade com a literatura, que associa baixa funcionalidade a baixos índices de qualidade de vida nessa população^2^^,^ 8 ^,^ 9.

Entre o tempo de realização do TUG e os domínios do Lymph-ICF-LL foram encontradas correlações positivas, sugerindo que quanto pior o desempenho no teste e lentidão para realizá-lo, maiores os escores dos domínios do questionário e, consequentemente, os prejuízos dessas funções em decorrência do linfedema.

Diante da utilização dos dois questionários, sendo um específico para o linfedema e outro genérico, confrontamos os domínios do Lymph-ICF-LL com aqueles do SF-36 que mais se adequavam às suas perguntas, a fim de analisar se eles correlacionavam entre si. Obtivemos correlações negativas altas e moderadas, demonstrando a aplicabilidade do Lymph-ICF-LL a essa população, ainda que ele não tenha sido validado no Brasil até o presente momento. Reforçamos os dados apresentados na literatura de que quanto maior a influência do linfedema na saúde do paciente, pior a qualidade de vida^7^^,^ 8, uma vez que quanto maior a pontuação obtida no Lymph-ICF-LL, menor a pontuação no SF-36.

Neste sentido, um maior enfoque na funcionalidade e na qualidade de vida desses indivíduos é necessário, uma vez que ambas se apresentaram influenciadas negativamente pela doença. Ressaltamos a aplicabilidade de instrumentos avaliativos como os questionários Lymph-ICF-LL, SF-36 e o teste TUG para a avaliação dessa população. Eles correlacionam entre si e possibilitam, através dos seus resultados, contribuições para a literatura científica e a prática clínica, que permitirão uma melhor compreensão do prognóstico e um melhor manejo da doença. Destacamos ainda o caráter precursor do estudo ao utilizar um instrumento específico recém disponibilizado no Brasil.

## CONCLUSÃO

O presente estudo evidencia que indivíduos com linfedema unilateral em membro inferior apresentam um impacto negativo na qualidade de vida e na funcionalidade relacionada às habilidades físicas, mentais e sociais avaliadas através de questionários, que correlacionam entre si. Embora não tenha sido encontrada alteração no tempo de realização do TUG, foi observado que este correlaciona os questionários SF-36 e Lymph-ICF-LL.

Sendo este estudo pioneiro na aplicação do Lymph-ICF-LL nessa população, novos estudos são necessários para comparar resultados e interpretar de forma mais robusta os achados desta pesquisa.
